# QSPR Studies on Aqueous Solubilities of Drug-Like Compounds

**DOI:** 10.3390/ijms10062558

**Published:** 2009-06-03

**Authors:** Pablo R. Duchowicz, Eduardo A. Castro

**Affiliations:** Instituto de Investigaciones Fisicoquímicas Teóricas y Aplicadas INIFTA (UNLP, CCT La Plata-CONICET), Diag. 113 y 64, C.C. 16, Suc.4, (1900) La Plata, Argentina; E-Mail: castro@quimica.unlp.edu.ar

**Keywords:** QSPR theory, aqueous solubility, ADME/Tox properties, Lipinski rules, molecular descriptors, replacement method, group contribution methods, high throughput screening techniques

## Abstract

A rapidly growing area of modern pharmaceutical research is the prediction of aqueous solubility of drug-sized compounds from their molecular structures. There exist many different reasons for considering this physico-chemical property as a key parameter: the design of novel entities with adequate aqueous solubility brings many advantages to preclinical and clinical research and development, allowing improvement of the Absorption, Distribution, Metabolization, and Elimination/Toxicity profile and “screenability” of drug candidates in High Throughput Screening techniques. This work compiles recent QSPR linear models established by our research group devoted to the quantification of aqueous solubilities and their comparison to previous research on the topic.

## 1. Introduction

Nowadays it is generally recognized that an ideal drug, besides being pharmacologically active, should additionally possess certain features regarding its bioavailability and its toxicological profile [1–5]. Absorption, Distribution, Metabolization, and Elimination/Toxicological (ADME/Tox) *in silico* filters constitute widely employed tools to determine whether it is probable or not for a drug candidate to reach its site of action or elicit toxic effects at its therapeutic dose. Moreover, modern approaches developed in the pharmaceutical industry for a rational molecular design have moved the ADME/Tox evaluations to the early stages of drug development, where an optimal activity of the compound is sought [6].

The degree of absorption of a substance depends simultaneously on dose, solubility, and permeability, and the exploration of large databases containing orally bioavailable drugs led to the formulation of the widely-used Lipinski “rule of five” for compounds absorbed through the gastrointestinal barrier via passive diffusion [7]. These simple rules state that oral bio-availability is likely to occur if at least three of the following rules are obeyed: molecular weight below 500; no more than five hydrogen bond donors and less than 10 hydrogen bond acceptors; and a calculated logarithm of the partition coefficient of the compound between water and octanol (log P) below 5.

The empirical conditions to satisfy Lipinski’s rule and display good oral bioavailability involve a balance between the aqueous solubility of a compound and its ability to diffuse passively through the different biological barriers. Aqueous solubility governs both the rate of dissolution of the compound and the maximum concentration reached in the gastrointestinal fluid. However, excessively polar compounds would result problematic at the stage of passing through the various biological barriers. Furthermore, it is known that aqueous solubility constitutes an important parameter in Medicinal Chemistry for the following reasons: soluble compounds are associated to shorter metabolization and elimination times, thus leading to lower probability of adverse effects and bioaccumulation [1,2,8], and most pre-clinical tests involve solubilization of the drug being tested in hydrophilic solvents [9,10]. Accurate activity measurements can be obtained only if the substance is sufficiently soluble (above the detection limit of the assay). Otherwise, an active compound may appear to be inactive due to insufficient solubility rather than inadequate potency [4,5].

The aqueous solubility of a given chemical entity can be obtained by experimental determination, although this usually presents some difficulties [2,3]. The traditional “shake flask” assay for measuring solubility is an equilibrium (thermodynamical) assay in which the solid is mixed vigorously with an aqueous buffer for a long period of time. This approach requires a fairly large amount of sample (1 – 2 mg) and is time-demanding (24 – 72 hours or more to do properly). Kinetic solubility measurements, in miniaturized methods such as Nephelometry [11], require little starting material but involve a reliable DMSO stock solution and multiple repeats to achieve accuracy. Furthermore, kinetic and thermodynamic solubility measurements are not interchangeable: they rely on fundamentally different physical properties to assess solid-state and solvation interactions and thus should be approached and interpreted with both caution and a detailed understanding of their strengths and limitations [12]. Obviously, it is not feasible to measure the solubility when no samples of compounds are available, while the times required for these assays are not compatible with the new High Throughput Screening technologies.

This background explains the great interest of developing theoretical models to predict aqueous solubility directly from structure. Consequently, a high number of theoretical models have been proposed in the past to predict aqueous solubilities, ranging from the early studies of Amidon *et al*. in 1975 [9] to several approaches including thermodynamic calculations, Group Contribution Methods and Quantitative Structure-Property Relationships (QSPR) [8,13–16].

## 2. Some Different *in silico* Methods for Solubility Estimation

The simplest definition for aqueous solubility (*S*, mol·L^−1^) in a given solvent is the maximum amount of the most stable crystalline form of the compound that can remain in solution in a given volume of the solvent at a given temperature and pressure under thermodynamic equilibrium [12]. This equilibrium balances the energy of the intermolecular interactions between solvent and solute molecules against the energy of solvent and solute molecules interacting intramolecularly with each other. For an ionizable compound, solubility without reference to pH and ionization constant pK_a_ is meaningless, while for any compound under analysis the specific solid state (amorphous or crystalline state) and solvent/s used is central for determining the solubility. It is also possible to distinguish different precise definitions of the term solubility [1].

The interaction between water and drug has been intensively studied previously and reviewed in ref. [2]. A typically employed empirical method to estimate solubility is based on easily obtained measurements, combining log *P* and melting point (MP) data by using the “General Solubility Equation” (GSE) [17–19]. Surprisingly, despite of its relative simplicity this equation has impressive accuracy as demonstrated in several studies [20–22], and this fact has led to the proposal of improved versions of the GSE model for adjusting large data sets of compounds [23–26]. The log *P* parameter provides an estimate of the strength of the interaction of the compound with water, while most common log *P* estimation programs are fragment based and empirical, such as CLOGP (Daylight Chemical Information Systems) and ACD/logD (Advanced Chemistry Development, Inc.). The main drawback of this method appears when it involves compounds having very high melting points (the sample decomposes before melting) or very low or very high log *P* values [15,27]. Other empirical methods were also reported, although sharing the common disadvantage that all of them require the experimental measurement of some terms defined in the equation [28,29].

The energetics of a compound in water can be assessed through a model of solvation, by resorting to Molecular Simulation in a statistical thermodynamical-like approach. Jorgensen and Duffy [30] employed Monte Carlo simulation with solute embedded in a bath of rigid water molecules to derive cohesive properties that can be used to predict solubility. However, this sort of calculations is quite computationally demanding for each different solute. A completely different approach to simulation is the Cellular Automata [31], were solvent and solute are represented by cells on a grid while their movements are governed by their immediate neighbors and a set of transition rules. The occupancy patterns of the cells change at each step, and many steps are involved. Such a kind of simulation offers intriguing insights into the dissolution process, i.e. formation of mobile cavities within the solid solute, but is not as useful as Monte Carlo in quantitative work. An alternative to the simulation of a large ensemble of particles focuses on a single solute molecule that is modeled in more detail, being based on electronic structure methods of Quantum Mechanics. Within this framework the solvent, which polarizes the molecule and is itself polarized by the solute, can be approximated as a continuous dielectric (Cramer-Truhlar approach) [32]. An alternative modeling of the solvent embeds both solute and solvent in a perfect conductor to calculate their polarization charge densities in the COSMO-RS (COSMOlogic GmbH and Co. KG) quantum chemical approach, leading to a chemical potential for the system that enables to estimate the solubility [33]. Despite of this, Quantum Mechanics methods are much slower than Monte Carlo simulations and result unsuitable for the analysis of large datasets of compounds. Table 1 summarizes different classes of methods to predict aqueous solubility data [1].

Among the different existing techniques for estimating different physical and thermodynamic data of interest, Group Contribution Methods (GCM) [34–36] are easy to apply, relying solely on the sum of contributions of each molecular structure fragment to the aqueous solubility. The basic assumption of this approach is the transferability concept for a group; if this hypothesis does not hold, then GCM can be corrected with experimental data when available to achieve better predictions. The methods proposed by Nirmalakhandan *et al*. [37], Suzuki *et al*. [38], Kuhne *et al*. [39], Lee *et al*. [40], and Klopman *et al*. [14,41] belong to this category. Among all these methods, only Klopman’s method is a pure and general group contribution model without using additional experimental parameters.

Although GCM have a simple and practical implementation, some common drawbacks of this methodology are the following: a) they require a large data set to obtain a contribution of each functional group; b) in its basic form (without corrections) it cannot model isomeric structures; c) they may contain a “missing fragment” problem, which means that if a compound contains a missing fragment which can be defined by the group contribution model, its aqueous solubility cannot be precisely predicted; d) there are not always measured data available to extend these methods to strange compounds such as molecules containing fused aromatic rings or to organometallic compounds. Since the final estimated GCM value assigned to the aqueous solubility of a compound involve that it change from the solid phase to a new one (liquid), this makes it harder to separate the contributions of individual parts of the molecule to the whole process. Nevertheless, GCM is a fast method for estimating aqueous solubility on large data sets of compounds and can produce reasonably accurate results.

## 3. Predicting Solubility through Linear Regression Based QSPR-QSAR

In the realms of the Quantitative Structure Property-Activity Relationships theory (QSPR-QSAR), a physicochemical or biological property of a compound is assumed to be a unique consequence of its molecular structure [42–44]. Therefore, a model is employed to predict the property by means of structural descriptors or numerical variables that capture different constitutional, topological, geometrical or electronic characteristics of the molecular structure in consideration. These molecular descriptors can be readily calculated through mathematical formulae obtained from several theories, such as the Chemical Graph Theory, Information Theory, Quantum Mechanics, etc. [45,46] The hypotheses involved in QSPR-QSAR analyzes have proven in the past to function quite well for a wide spectrum of properties/activities of interest.

QSPR-QSAR models enable property estimation for substances that have yet not been tested for different reasons, such as instability, toxicity, or simply because their measurement requires too much time. In terms of economy, these studies allow the rational use of the available resources present in the laboratory or even a plant, avoiding performing expensive and unnecessary experimental determinations. With respect to their moral aspects, the QSPR-QSAR analyses applied to Toxicology have achieved great importance in the virtual screening of the toxic potential of compounds before their synthesis [47], and thus represent an effective alternative that reduces animal testing in biological assays. In drug discovery, both the prediction with QSAR-QSPR of ADMET properties [48] and the oral bioavailability of compounds [49,50] were conveniently addressed. Finally, from the theoretically point of view, the model can illuminate the mechanisms of physicochemical properties or biological activities of the compounds.

It is well known that a single descriptor is unable to carry all the structural information of a molecule, and one has to search for the best descriptors among the more than a thousand available in the literature, that are the most representative/descriptive parameters for the particular modeled property [51–53]. There exist various standard statistical methods that constitute a common practice for QSPR-QSAR model design, such as linear: Multivariable Linear Regression (MLR) [54], Principal Component Analysis (PCA) [55], Genetic Algorithms [56], Replacement Method [57], and non-linear methods: Artificial Neural Networks (ANN) [58], or Support Vector Machines [59]. The main advantage of developing linear models compared to non-linear ones is the fact that the former suffer less from the over-fitting (over-training) problem [60,61], they are more general and can transparently reveal the effect of the structural variables present in the model upon the property being modeled, thus making it possible to suggest cause/effect relationships.

## 4. The Proposal of Descriptors Based on Lipinski Rules for Modeling Aqueous Solubilities

One of our recent QSPR studies analyzing aqueous solubilities employs MLR for establishing the connection between the solubility values of 148 heterogeneous organic chemicals and their molecular structure, represented through a new set of physically interpretable descriptors [62]. The correct representation of the molecular structure of drug like compounds through molecular descriptors in every QSPR-QSAR study is of crucial importance. The set of descriptors introduced here is characterized by involving in a single number several of the parameters described by the Lipinski rules [7]. The proposed Lipinski based descriptors are based on combinations of the detour index (*dd*) from Chemical Graph Theory (derived as the half sum of the elements of the Detour Matrix - *DD*) [63] together with molecular features such as the number of H donors (*D*), the number of H acceptors (*A*) and the number of heteroatoms (*H*) present in the structure:

(1) $$D\;/\;D=\frac{dd}{D+0.1}\;\;D\;/\;A=\frac{dd}{A}\;\;D\;/\;B=\frac{dd}{A+D}\;\;D\;/\;H=\frac{dd}{H}$$

where the 0.1 term in the *D/D* definition is introduced only to prevent dividing by zero, considering that several of the studied compounds do not have any H donor functional group.

The above descriptor definitions take into consideration many literature reports which demonstrate linear, polynomial and exponential correlations between *dd* and the boiling point of alkanes, cycloalkanes and aromatic compounds [64–68]. Since the boiling point of compounds from homologous series usually correlates well with molecular weight (MW), we have investigated the relationship between the *dd* and the MWs of the 148 compounds used for the present study. Inspection of the correlation between *dd* and MW pushed us to explore possible relationships between the square and cubic roots of *dd* and the MW. It is noticeable that cubic root of *dd*, in the first place, and square root of *dd*, in the second, display quite better linear correlations with the molecular weight of the 148 structures (*R* = 0.932 and *R* = 0.918, in that order). This is an indication of very good correlation, specially noticing the structural diversity of the dataset.

It is clear then that the Detour Index may be an appropriate descriptor to explain the differences in the aqueous solubility values that could be explained through the molecular weight of compounds. It can also characterize other molecular properties such as the degree of ramification and cyclization. However, there are a lot of examples of compounds that, although sharing the same graph and therefore the same *dd* value, have very different solubilities because of the other three parameters included in Lipinski’s rule (number of H donor and acceptors and log *P*). To answer this issue we have included *A*, *B* (= *A* + *D*), and *H* in the new descriptor’s definition. We also considered the square and cubic roots of the four descriptors above (*D*/*D*^1/2^, *D*/*D*^1/3^, *D*/*A*^1/2^, *D*/*A*^1/3^, *D*/*B*^1/2^, *D*/*B*^1/3^, *D*/*H*^1/2^, and *D*/*H*^1/3^), based on the better correlation between the squares and cubic roots of *dd* and MW compared to that between *dd* and MW. The physicochemical meaning of these descriptors is immediate. MW is directly correlated with *dd*, and the solubility tends to decrease, in homologous series, when MW increases. The more H donor and acceptors present in the molecule the more water soluble the compound will be. If no H donor or acceptor is present in the molecule, the water solubility would be jeopardized or even non existent (as is the case of alkanes). Therefore, the defined descriptors will take high values in compounds with slight aqueous solubility, while they will tend to infinite in non-soluble compounds.

We proceeded to search for a QSPR solubility model that minimizes the *S* parameter subjected to the condition of combining at least one of the proposed molecular descriptors reflecting the Lipinski rules together with those calculated with the Dragon software [69]. The application of the Replacement Method (RM) variable subset selection technique [57,70,71] to the available pool with *D* = 1,367 descriptors leads to an optimal relationship over 100 compounds that, in terms of the best predictive power of the equation measured via the calibration and the *l-n%-o* parameters [72] and the least number of variables involved, contains six molecular descriptors of different type:

(2) $$\begin{array}{l} {\text{log}}_{10}Sol=2.786(\pm 0.3)\;+\;0.0479(\pm 0.02)\;\mathrm{RDF040e}\;+\;0.285(\pm 0.07)\;C-006-5.639(\pm 0.7)\\ \;\;\;\;H3p\;+\;0.00389\;(\pm 0.001)\;D/A\;-\;0.231(\pm 0.04)\;D/B^{1/2}\;+\;0.00988(\pm 0.002)\;\;QXXe\\ \;\;\;\;\;\;\;N=100,\;R=0.880,\;S=0.858,\;F=53.091,\;p\;<\;10^{-4},\\ \;\;\;\;\;\;R_{loo}=0.853,\;S_{loo}=0.911,\;R_{l-10\%-o}=,0.820,\;S_{l-10\%-o}=1.006. \end{array}$$

where the absolute errors of the regression coefficients are given in parentheses and *R* is the correlation coefficient, *F* is the Fisher ratio and *p* is the significance of the model. Quite good estimations can be achieved with this QSPR model in many cases, considering the heterogeneous nature of the training set of molecules extracted from Merck Index 13^th^ [73]. About 99% of these compounds are “drug-like”, satisfying Lipinski’s rule.

Equation (2) involves different molecular descriptors that can be classified as follows: two of the proposed absorption-based descriptors: *D*/*A* and *D*/*B*^1/2^; a Radial Distribution Function (RDF): *RDF040e*, RDF-4.0/weighted by atomic Sanderson electronegativities [74]; a GETAWAY descriptor: *H3p*, H autocorrelation of lag 3/weighted by atomic polarizabilities [75]; an Atom-Centred Fragment: *C-006*, the number of CH_2_RX functional groups [X: heteroatom (O, N, S, P, Se or halogens), R: any group linked through carbon] [76]; and a geometrical descriptor: *QXXe*, Qxx COMMA2 value/weighted by atomic Sanderson electronegativities [77]. A next step in the present analysis was to further validate the predictive power of the QSPR solubility model found by predicting the log(*Sol*) values in a test set containing 48 organic compounds, thus demonstrating that it is possible to achieve good estimations in many situations.

## 5. A QSPR Designed upon a Balanced Aqueous Solubility Data Set

It has been pointed out that solubility modeling efforts have suffered from some basic concerns, among them: training sets that are not drug-like, lack of structural diversity, unknown experimental error, incorrect tautomers or structures, neglect of ionization and crystal packing effects, over-sampling of compounds with low molecular weight and range in solubility data that is not pharmaceutically relevant [2,4]. Another study conducted by our research group [78] tries to answer some of the previous issues, since it is developed from a structural diverse training set composed by drug-like compounds with more than half the dataset presenting solubility values below 1 mg·mL^−1^. Note that low solubility compounds are actually the ones one would like to be able to predict accurately, since they have higher probability of presenting difficulties in pre-clinic and clinic assays and formulation stages. Therefore, the QSPR Theory was employed for analyzing the aqueous solubility exhibited at 298 K by 145 diverse drug-like organic compounds. The molecular set was split into a 97-compound training set (train) and a 48-compounds test set (val), selecting the members of each set in such a way to share similar structural characteristics of the compounds. Additionally, an external molecular set (test set 21) that was not involved during the model design, and composed of 21 well-known compounds found in many solubility prediction papers, was also employed [2,14], in order to further examine the model’s validation.

**Figure 1.**
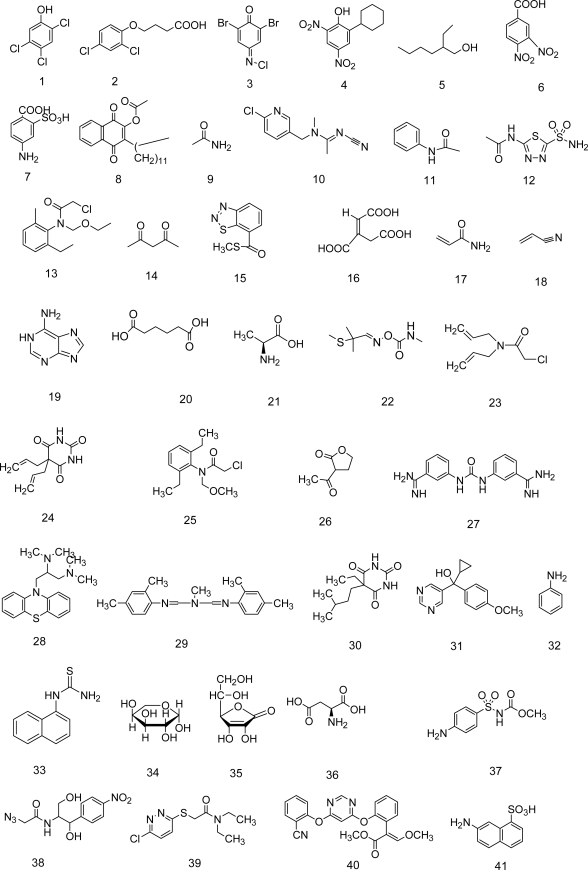

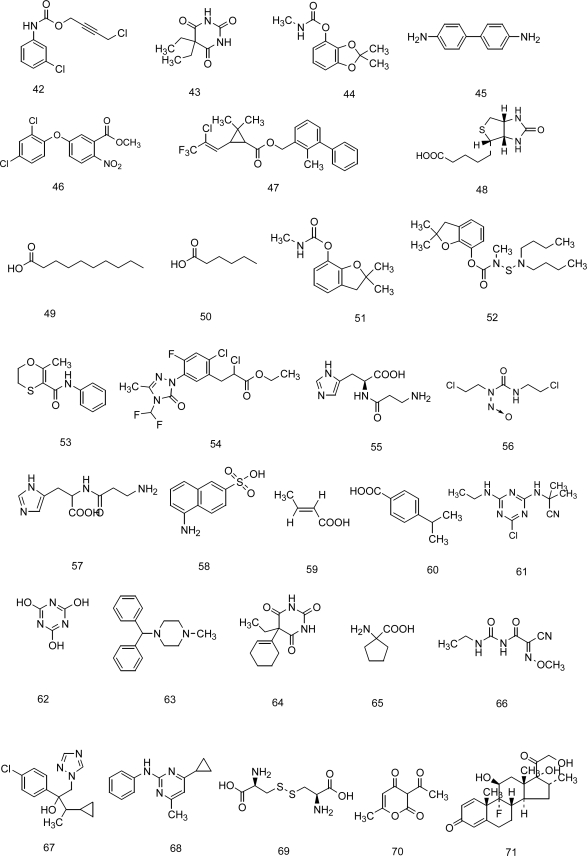

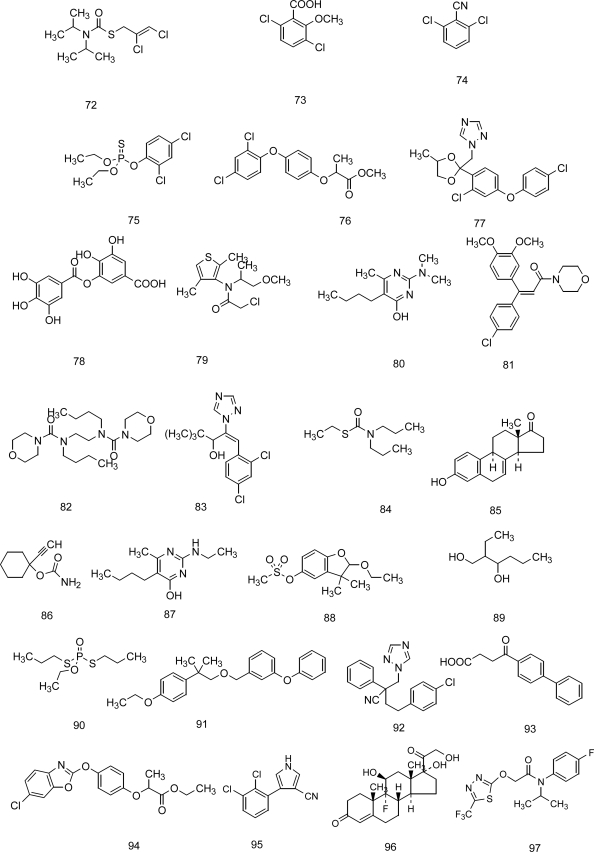

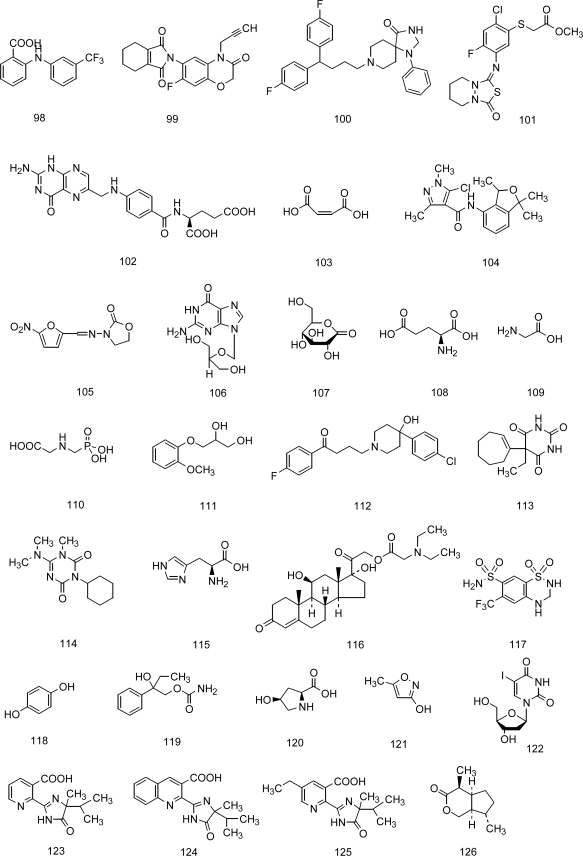

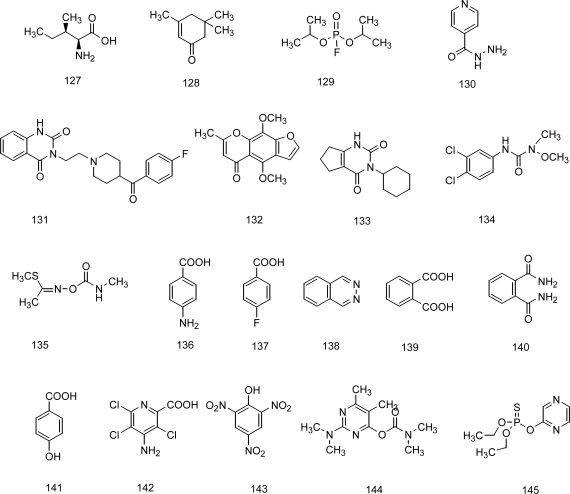
Balanced data set of molecular structures under analysis. Training Set 1–97 Test Set 98–145.

In this work, most of the drugs that comprise the training and test sets meet several drug-likeness criteria. More than 99% of the data set observes the Lipinski-rule criteria for estimating drug oral bioavailability [7], while more than 93% fulfill the Veber *et al*. rule [79]. More than 99% of the dataset also meets more general criteria for evaluating drug-likeness extracted from several recent publications: [80–82] 100 ≤ molecular weight ≤ 800 g·mol^−1^; log *P* ≤ 7; number of H bond acceptors ≤ 10; number of H bond donors ≤5; rotatable bonds ≤15; halogen atoms ≤7; alkyl chains ≤ (CH_2_)_6_CH_3_; no perfluorinated chains: CF_2_CF_2_CF_3_; no large rings (i.e. with more than seven members); no presence of atoms other than C, O, N, S, P, F, Cl, Br, I, Na, K, Mg, Ca or Li and; presence of at least one N or O atom. Moreover, low molecular weight compounds are not over-represented in this molecular set. All the molecular structures are drawn in Figure 1.

**Figure 2.**
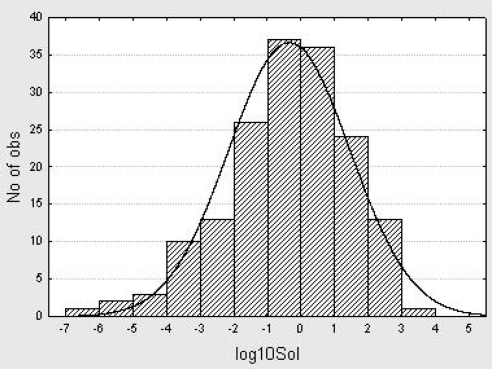
Normal distribution of the experimental log_10_*Sol* values under analysis (*N* = 166).

The structural diversity of the training set is assessed through calculation of the average Tanimoto intermolecular distances (based on atom pairs) for all the possible pairs of structures that could be derived from the training set. For this purpose the PowerMV software provided by the National Institute of Statistical Sciences was used [83]. According to the results, the average Tanimoto intermolecular distance for the training set is 0.781 with a *S* of 0.412, which confirms the high structural diversity of the training set. Figure 2 shows a histogram representing the distribution of the 166 aqueous solubilities under study, which suggests that the experimental sample is normally distributed over more than four logarithmic units and can thus be employed in regression analysis.

The initial conformations of the drug compounds are obtained by means of the “model build” modulus of the HyperChem package [84]. After that, the structures of the compounds are firstly pre-optimized with the Molecular Mechanics Force Field (MM+) procedure included in the Hyperchem, and the resulting geometries are further refined by means of the Semi-Empirical Method PM3. More than a thousand DRAGON [69] theoretical descriptors are simultaneously explored including definitions of all classes, by means of the linear variable subset selection approach Replacement Method (RM) [57, 70, 71]. The application of the RM method on the training set of 97 heterogeneous drugs leads to the following satisfactory three-descriptors relationship:

(3) $$\begin{array}{l} {\text{log}}_{10}Sol=-0.435(\pm 0.03)\;\cdot \;\Omega (X1sol)-0.503(\pm 0.06)\cdot \Omega (MLOGP)+\\ \;\;\;\;\;0.0767(\pm 0.01)\cdot \Omega (RDF060u)\;+\;2.970(\pm 0.3)\\ N_{train}=97,\;N_{train}/d=32.333,\;R=0.871,\;S=0.903,\;R_{loo}=0.849,\;S_{loo}=0.971,\\ R_{l-10\%-o}=0.809,\;S_{l-10\%-o}=1.090,\;p\;<\;10^{-4},\;N_{val}=48,\;R_{val}=0.848,\;S_{val}=0.899 \end{array}$$

The QSPR derived does not incorporate redundant structural information, as it involves orthogonal descriptors [85]. This model includes two calibration outliers with a residual exceeding the value 2*S* = 1.806: compounds **15** (acibenzolar-*S*-methyl, 1.902) and **91** (etofenprox, −2.545), while no one of the training compounds exceed the value 3*S* = 2.709; the presence of these outliers may be attributed exclusively to be a pure consequence of the limited number of structural descriptors participating in Eq. (3), since this model haa a high ratio of number of observations to number of parameters (*N*/*d* = 32.333).

The predictive power of the QSPR is satisfactory, as revealed by its stability upon the inclusion or exclusion of compounds, as measured by the *loo* parameters *R_loo_* = 0.849 and *S_loo_* = 0.971, and by the more severe test of higher percentage of compounds exclusion *R_l-10%-o_* = 0.809 and *S_l-10%-o_* = 1.090. These results are in the range of a validated model: *R_l-n%-o_* must be greater than the value of 0.50, according to the specialized literature [86]. Furthermore, the predictive capability of the so established equation is demonstrated by its performance in the test set val, leading to *R_val_* = 0.848 and *S_val_* = 0.899. Finally, after analyzing 5,000,000 cases for y-randomization [87], the smallest *S* value obtained using this procedure was 1.650, a poorer value when compared to the one found considering the true calibration (*S* = 0.903). In this way, the robustness of the model could be assessed, showing that the calibration was not a fortuitous correlation and therefore results in a structure-activity relationship.

As can be appreciated from the derived QSPR, different definitions of descriptors are needed to correctly represent the structures for the drug-like heterogeneous compounds. After a proper standardization [88] of the orthogonal descriptors present in Equation (3), it is feasible to assign a greater importance to those variables that exhibit larger absolute standardized coefficients. The most important structural factor of the model is the topological descriptor *X1sol*, the solvation connectivity index chi-1 proposed by Zefirov and Palyulin in 1991 [89]. It has the following general formula when calculated for hydrogen- and fluorine-depleted molecular graphs:

(4) $$Xmsol=(1/2^{m+1})\sum \frac{Z_{i}Z_{j}\ldots Z_{k}}{{\left(\delta_{i}\delta_{j}\ldots \delta_{k}\right)}^{1/2}}$$

where *m* is the order of index; summation is over all sub-graphs of order *m; δ_i_δ_j_* ...*δ_k_* are connectivities of vertexes of sub-graph; and *Z_i_Z_j_* ...*Z_k_* are coefficients characterizing the atom size, which coincide to the number of the period in the Periodic Table. The second important descriptor involved in Eq. (3) corresponds to *MLOGP*, the Moriguchi octanol-water partition coefficient [90]: this reveals that a compound’s hydrophobicity plays a crucial role in explaining the aqueous solubility data. Finally, the contribution of a 3D-Radial Distribution Function [74] *RDF060u* helps to improve the predictive power of the QSPR. Such a kind of molecular descriptor defined for an ensemble of atoms may be interpreted as the probability distribution of finding an atom in a spherical volume of certain radius, incorporating different types of atomic properties in order to differentiate the nature and contribution of atoms to the property being modelled. For the case of *RDF060u*, the sphere radius is of 6.0 angstroms and no atomic property is employed, thus characterizing the molecular size.

The application of the developed structure-property relationship to the classical test set 21, whose data are considered “unknown” and that do not participate during the model development (as is the case of test set val), leads to a square root mean quadratic residual (*rms*) of 1.202. The statistical quality achieved on this test set is comparable to that obtained by the previously reported models for aqueous solubilities in Table 2, and the main advantage here is that only three molecular descriptors are employed to model the physical property, leading to a favorable ratio *N*/*d* = 7. This equation results in a superior predictive quality than that obtained by the GCM of Klopman (*rms* = 1.213) involving 34 parameters [14], and also outperforms the MLR of Yan (*rms* = 1.286) using 40 parameters [91].

To conclude the present analysis, the chemical information encoded by only three theoretical molecular descriptors of the one-, two-, and three- types participating in a linear QSPR model enabled to explain the variation of the experimental aqueous solubilities in a satisfactory extent, and allowed a proper characterization of structurally heterogeneous drug-like organic compounds from both the training and test sets. The QSPR designed involved molecular descriptors that have a quite direct interpretation, and this relationship proved to have general applicability. The statistical parameters of the proposed model compare fairly well with others published previously based on the GCM methodology.

## 4. Conclusions

In this review we have analyzed the possibility of establishing quantitative structure-aqueous solubility relationships for drug-like compounds, and compared our recently developed linear QSPR method with others reported in the literature. Such kinds of linear equations are demonstrated to work quite well both for the training and validation stages of the model, and can in principle be used for the *in silico* prediction of physicochemical properties. Two different strategies can be adopted for correlating the structure and the solubility of compounds: (a) the proposal of novel descriptors posing some kind of physical interpretation, as it is the case for the Lipinski’s “rule of five” descriptors taking into account the bio-availability of drugs, or (b) the use of any kind of constitutional, topological, geometrical, or electronic descriptors for adjusting to the experimental solubility data. In both cases, it results of considerable importance the appropriate selection of a balanced set of chemical compounds that considers structural diversity, known experimental errors, correct tautomers or structures, consideration of ionization and crystal packing effects, range in solubility data that is pharmaceutically relevant, and that avoid the over-sampling of compounds with low molecular weight.

## Figures and Tables

**Table 1.** Methods for predicting aqueous solubilities.

**Description**	**Requirements**	**Speed**
Methods based on other experimental physico-chemical properties	log *P*, MP, etc.	Tens to hundreds compounds per day
Methods using 3D parameters depending on molecular stereochemistry	Optimized 3D structure, Monte Carlo, quantum chemical calculations	Tens to tens of thousands compounds per day
Fragmental and atom-type based methods using 1D or 2D parameters	Molecule as a smile, 2D graph	Million of compounds per day

**Table 2.** Performance of different linear methods applied on the same 21-test set compounds.

**Lead author**	**Method**	**Type of descriptors**	**Number of parameters**	**rms**	**N/d**	**Reference**	**Year**
Klopman	GCM	2D Substructures	34	1.213	0.62	[14]	1992
Yan	MLR	3D Descriptors	40	1.286	0.53	[91]	2003
Hou	GCM	Atomic	78	0.664	0.27	[92]	2004
Huuskonen	MLR	Topologicals	30	0.810	0.70	[93]	2000
Duchowicz	MLR	Dragon	3	1.202	7.00	this study	2008

## References

[1] Balakin KV Savchuk NP Tetko IV In Silico approaches to prediction of aqueous and DMSO Solubility of drug-like compounds: Trends, problems and solutions Curr. Med. Chem 2006 13 226 241 10.2174/092986706775197917 16472214

[2] Delaney JS Prediction of aqueous solubility from structure Drug Disc. Today 2005 10 289 295 10.1016/S1359-6446(04)03365-3 15708748

[3] Goodwin JJ Rationale and benefit of using high throughput solubility screens in drug discovery Drug Disc. Today Technol 2006 3 67 71 10.1016/j.ddtec.2005.03.001 24980103

[4] Johnson SR Zheng W Recent progress in the computational prediction of aqueous solubility and absorption AAPS J 2006 8 E27 E40 16584131 10.1208/aapsj080104 PMC2751421

[5] Schneider G So S Adaptative Systems in Drug Design Landes Bioscience Austin, TX, USA 2003

[6] Yu H Adedoyin A ADME-Tox in drug discovery: integration of experimental and computational technologies Drug Disc. Today 2003 8 852 861 10.1016/s1359-6446(03)02828-9 12963322

[7] Lipinski CA Lombardo F Dominy DW Feeney PJ Experimental and computational approaches to estimate solubility and permeability in drug discovery and development settings Adv. Drug Deliv. Rev 2001 46 3 26 11259830 10.1016/s0169-409x(00)00129-0

[8] Smith CJ Hansch C The relative toxicity of compounds in mainstream cigarette smoke condensate Food Chem. Toxicol 2000 38 637 646 10942325 10.1016/s0278-6915(00)00051-x

[9] Amidon GL Yalkowsky SH Anik ST Valvani SC Solubility of nonelectrolytes in polar solvents. V. Estimation of the solubility of aliphatic monofunctional compounds in water using a molecular surface area approach J. Phys. Chem. A 1975 79 2239 2246

[10] Hansch C Bjorkroth JP Leo A Hydrophobicity and central nervous system agents: on the principle of minimal hydrophobicity in drug design J. Pharm. Sci 1987 76 663 687 11002801 10.1002/jps.2600760902

[11] Kariv I Rourick RA Kassel DB Chung TD Improvement of “hit-to-lead” optimization by integration of *in vitro* HTS experimental models for early determination of pharmacokinetic properties Comb. Chem. High Throughput Screen 2002 5 459 472 12470275 10.2174/1386207023330101

[12] Bhattachar SN Deschenes LA Wesley JA Solubility: it's not just for physical chemists Drug Disc. Today 2006 11 1012 1018 10.1016/j.drudis.2006.09.002 17055411

[13] Katritzky AR Maran U Lobanov VS Karelson M Structurally diverse quantitative structure-property relationship correlations of technologically relevant physical properties J. Chem. Inf. Model 2000 40 1 18 10.1021/ci9903206 10661545

[14] Klopman G Wang S Balthasar DM Estimation of aqueous solubility of organic molecules by the group contribution approach. Application to the study of biodegradation J. Chem. Inf. Model 1992 32 474 482 10.1021/ci00009a013 1400663

[15] McFarland JW Avdeef A Berger CM Raevsky OA Estimating the water solubilities of crystalline compounds from their chemical structure alone J. Chem. Inf. Model 2001 41 1355 1359 10.1021/ci0102822 11604037

[16] Pogliani L Modeling purines and pyrimidines with the linear combination of connectivity indices–molecular connectivity “LCCI-MC” method J. Chem. Inf. Model 1996 36 1082 1091

[17] Yalkowsky SH Valvani SC Solubility and partitioning I: solubility of nonelectrolytes in water J. Pharm. Sci 1980 69 912 922 7400936 10.1002/jps.2600690814

[18] Yalkowsky SH Valvani SC Roseman TJ Water solubility: A critique of the solvatochromic approach J. Pharm. Sci 1983 72 866 870 6620139 10.1002/jps.2600720808

[19] Yang G Ran Y Yalkowsky SH Prediction of the aqueous solubility: comparison of the general solubility equation and the method using an amended solvation energy relationship J. Pharm. Sci 2002 91 517 533 11835210 10.1002/jps.10022

[20] Peterson DL Yalkowski SH Comparison of two methods for predicting aqueous solubility J. Chem. Inf. Comput. Sci 2001 41 1531 1534 11749579 10.1021/ci010298s

[21] Ran Y Yalkowsky SH Prediction of drug solubility by the general solubility equation (GSE) J. Chem. Inf. Comput. Sci 2001 41 354 357 11277722 10.1021/ci000338c

[22] Ran Y Jain N Yalkowsky SH Prediction of aqueous solubility of organic compounds by the general solubility equation (GSE) J. Chem. Inf. Comput. Sci 2001 41 1208 1207 11604020 10.1021/ci010287z

[23] Meylan WM Howard PH Boethling RS Improved method for estimating water solubility from octanol/water coefficient Environ. Toxicol. Chem 1996 15 100 106

[24] Meylan WM Howard PH Estimating log P with atom/fragments and water solubility with log P Persp. Drug Disc. Design 2000 19 67 84

[25] Myrdal P Ward GH Dannenfelser RM Mishra DS Yalkowsky SH AQUAFAC 1: Aqueous Functional group activity coefficients: Application to hydrocarbons Chemosphere 1992 24 1047 1061

[26] Pinsuwan S Myrdal PB Lee YC Yalkowsky SH AQUAFAC 5: Applications to alcohols and acids Chemosphere 1997 35 2503 2513

[27] Morris JJ Bruneau PP Prediction of physicochemical properties Virtual Screening for Bioactive Molecules Bohm HG Schneider G Wiley-VCH Weinheim, Germany 2000 10 33 58

[28] Thompson JD Cramer CJ Truhlar DG Predicting aqueous solubilities from aqueous free energies of solvation and experimental or calculated vapor pressures of pure substances J. Chem. Phys 2003 119 1661 1670

[29] Yaws CL Xiang P Xiaoyin L Water solubility data for 151 hydrocarbons Chem. Eng 1993 100 108 111

[30] Jorgensen WL Duffy EM Prediction of drug solubility from Monte Carlo simulations Bioorg. Med. Chem. Lett 2000 10 1155 1158 10866370 10.1016/s0960-894x(00)00172-4

[31] Kier LB Cheng C-K Seybold PG Cellular automata models of aqueous solution systems Reviews in Computational Chemistry Lipkowitz KB Boyd DB Wiley-VCH Weinheim, Germany 2001 17 205 254

[32] Cramer CJ Truhlar DG Continuum solvation models: Classical and quantum mechanical implementations Reviews in Computational Chemistry Lipkowitz KB Boyd DB Wiley-VCH Weinheim, Germany 1995 6 1 72

[33] Klamt A Prediction of aqueous solubility of drugs and pesticides with COSMO-RS J. Comput. Chem 2002 23 275 281 11924739 10.1002/jcc.1168

[34] ArtistAvailable online: http://www.ddbst.de/new/Win_DDBSP/frame_Artist.htm, 2 June 2009.

[35] ChemEng Software DesignAvailable online: http://www.cesd.com/chempage.htm, 2 June 2009.

[36] PredictAvailable online: http://www.mwsoftware.com/dragon/desc.html, 2 June 2009.

[37] Nirmalakhandan NNP Speece RE Prediction of aqueous solubility of organic chemicals based on molecular structure. 2. Application to PNAs, PCBs, PCDDs, etc Environ. Sci. Technol 1989 23 708 713 10.1021/es00168a014 22288868

[38] Suzuki T Development of an automatic estimation system for both the partition coefficient and aqueous solubility J. Comput.-Aided Mol. Des 1991 5 149 166 1869898 10.1007/BF00129753

[39] Kuhne R Ebert RU Kleint F Schmidt G Schuurmann G Group contribution methods to estimate water solubility of organic chemicals Chemosphere 1995 30 2061 2077

[40] Lee Y Myrdal PB Yalkowsky SH Aqueous functional group activity coefficients (AQUAFAC) 4: Applications to complex organic compounds Chemosphere 1996 33 2129 2144

[41] Klopman G Zhu H Estimation of aqueous solubility of organic molecules by the group contribution approach J. Chem. Inf. Model 2001 41 439 445 10.1021/ci000152d 11277734

[42] Free SM Wilson JW A mathematical contribution to structure-activity studies J. Med. Chem 1964 7 395 399 14221113 10.1021/jm00334a001

[43] Hansch C p-σ-π analysis. A method for the correlation of biological activity and chemical structure J. Am. Chem. Soc 1964 86 1616 1626

[44] Hansch C Leo A Exploring QSAR Fundamentals and Applications in Chemistry and Biology American Chemical Society Washington, DC, USA 1995

[45] Katritzky AR Lobanov VS Karelson M QSPR - the correlation and quantitative prediction of chemical and physical properties from structure Chem. Soc. Rev 1995 24 279 287

[46] Trinajstic N Chemical Graph Theory CRC Press Boca Raton, FL, USA 1992

[47] Worth AP Bassan A De Bruijn J Saliner AG Netzeva T Patlewicz G Pavan M Tsakovska I Eisenreich S The role of the European Chemicals Bureau in promoting the regulatory use of QSARs methods SAR QSAR Environ. Res 2007 18 111 125 17365963 10.1080/10629360601054255

[48] Noringer U In silico modelling of ADMET-a minireview of work from 2000 to 2004 SAR QSAR Environ. Res 2005 16 1 11 10.1080/10629360412331319835 15844440

[49] Martin YC A bioavailability score J. Med. Chem 2005 48 3164 3170 15857122 10.1021/jm0492002

[50] Yoshida F QSAR model for drug human bioavailability J. Med. Chem 2000 43 2575 2585 10891117 10.1021/jm0000564

[51] Molecular Descriptors Family Home pageAvailable online: http://sorana.academicdirect.ro, 2 June 2009.

[52] Karelson M Molecular Descriptors in QSAR/QSPR Wiley-Interscience New York, NY, USA 2000

[53] Todeschini R Consonni V Handbook of Molecular Descriptors Wiley VCH Weinheim, Germany 2000

[54] Apostol TM Calculus Blaisdell Publishing Co Waltham, MA, USA 1969

[55] Malinowski ER Factor Analysis in Chemistry Wiley New York, NY, USA 1991

[56] Leardi R Genetic algorithms in feature selection Genetic Algorithms in Molecular Modeling Principles of QSAR and Drug Design Devillers J Academic Press London, UK 1996 1 67 86

[57] Duchowicz PR Castro EA Fernández FM Alternative algorithm for the search of an optimal set of descriptors in QSAR-QSPR studies MATCH Commun. Math. Comput. Chem 2006 55 179 192

[58] Zupan J Encyclopedia of Computational Chemistry Wiley Chichester, UK 19982006

[59] Vapanik V The Nature of Statistical Learning Theory Springer Verlag New York, NY, USA 1995

[60] Livingstone DJ Manallack DT Statistics using neural networks: chance effects J. Med. Chem 1993 36 1295 1297 8487267 10.1021/jm00061a023

[61] Tetko IV Luik AI Poda GI Applications of neural networks in structure-activity relationships of a small number of molecules J. Med. Chem 1993 36 811 814 8464034 10.1021/jm00059a003

[62] Talevi A Castro EA Bruno-Blanch LE New solubility models based on descriptors derived from the detour matrix J. Arg. Chem. Soc 2006 44 129 141

[63] Harary F Graph Theory Addison-Wesley Upper Saddle River, NJ, USA 1969

[64] Castro EA Tueros M Toropov AA Maximum topological distances based indices as molecular descriptors for QSPR: 2--application to aromatic hydrocarbons Comput. Chem 2000 24 571 576 10890366 10.1016/s0097-8485(99)00095-9

[65] Devillers J Balaban AT Topological Indices and Related Descriptors in QSAR and QSPR Gordon and Breach Science Publishers New York, NY, USA 1999

[66] Firpo M Gavernet L Castro EA Toropov AA Maximum topological distances based indices as molecular descriptors for QSPR. Part 1. Application to alkyl benzenes boiling points J. Mol. Struc-Theochem 2000 501 419 425

[67] Lukovits I The detour index Croat. Chem. Acta 1996 69 873 882

[68] Trinajstić N Nikolić S Lučić B The detour matrix in chemistry J. Chem. Inf. Model 1997 37 631 638

[69] Milano Chemometrics and QSAR Research Group HomepageAvailable online: http://www.disat.unimib.it/chm, 2 June 2009.

[70] Duchowicz PR Castro EA Fernández FM González MP A new search algorithm of QSPR/QSAR theories: Normal boiling points of some organic molecules Chem. Phys. Lett 2005 412 376 380

[71] Duchowicz PR Fernández M Caballero J Castro EA Fernández FM QSAR of non-nucleoside inhibitors of HIV-1 reverse transcriptase Bioorg. Med. Chem 2006 16 5876 5889 16766190 10.1016/j.bmc.2006.05.027

[72] Hawkins DM Basak SC Mills D Assessing model fit by cross validation J. Chem. Inf. Model 2003 43 579 586 10.1021/ci025626i 12653524

[73] The Merck Index An Encyclopedia of Chemicals, Drugs, and Biologicals13th Ed Merck & Co Rahway, NJ, USA 2001

[74] Consonni V Todeschini R Pavan M Structure/response correlations and similarity/diversity analysis by GETAWAY descriptors. 2. Application of the novel 3D molecular descriptors to QSAR/QSPR studies J. Chem. Inf. Model 2002 42 693 705 10.1021/ci0155053 12086531

[75] Consonni V Todeschini R Rational Approaches to Drug Design Prous Science Barcelona, Spain 2001 235 240

[76] Viswanadhan VN Ghose AK Revankar GR Robins RK Atomic physicochemical parameters for three dimensional structure directed quantitative structure-activity relationships. 4. Additional parameters for hydrophobic and dispersive interactions and their application for an automated superposition of certain naturally occurring nucleoside antibiotics J. Chem. Inf. Model 1989 29 163 172

[77] Silverman DB Three-dimensional moments of molecular property fields J. Chem. Inf. Model 2000 40 1470 1476 10.1021/ci000457s 11128106

[78] Duchowicz PR Talevi A Bruno-Blanch LE Castro EA New QSPR study for the prediction of aqueous solubility of drug-like compounds Bioorg. Med. Chem 2008 16 7944 7955 18701302 10.1016/j.bmc.2008.07.067

[79] Veber DF Johnson SR Cheng H Smith BR Ward KW Kopple KD Molecular property that influence the drug bioavailability of drug candidates J. Med. Chem 2002 45 2615 2623 12036371 10.1021/jm020017n

[80] Charifson PS Walters WP Filtering databases and chemical libraries J. Comput. Aided Mol. Des 2002 16 311 323 12489681 10.1023/a:1020829519597

[81] Monge A Arrault A Marot C Morin-Allory L Managing, profiling and analyzing a library of 2.6 million compounds gathered from 32 chemical providers Mol. Divers 2006 10 339 403 10.1007/s11030-006-9033-5 17031540

[82] Walters WP Murcko MA Prediction of “drug-likeness” Adv. Drug Deliv. Rev 2002 54 255 271 11922947 10.1016/s0169-409x(02)00003-0

[83] Liu K Feng J Young SS PowerMV: A software environment for molecular viewing, descriptor generation, data analysis and hit evaluation J. Chem. Inf. Model 2005 45 515 522 15807517 10.1021/ci049847v

[84] Hyperchem (Hypercube) HomepageAvailable online: http://www.hyper.com, 2 June 2009.

[85] Randic M Resolution of ambiguities in structure-property studies by use of orthogonal descriptors J. Chem. Inf. Model 1991 31 311 320

[86] Golbraikh A Tropsha A Beware of q2! J. Mol. Graphics Model 2002 20 269 276 10.1016/s1093-3263(01)00123-1 11858635

[87] Wold S Eriksson L Chemometrics Methods in Molecular Design VCH Weinheim, Germany 1995

[88] Draper NR Smith H Applied Regression Analysis John Wiley & Sons New York, NY, USA 1981

[89] Antipin IS Arslanov NA Palyulin VA Konovalov AI Zefirov NS of Disperse Interactions Dokl Akad Nauk SSSR 1991 316 925 928 (*Chem. Abstr. 115*, 91390).

[90] Moriguchi I Hirono S Liu Q Nakagome I Matsuchita Y Simple method of calculating octanol/water partition coefficient Chem. Pharm. Bull 1992 40 127 130

[91] Yan A Gasteiger J Prediction of aqueous solubility of organic compounds based on a 3D structure representation J. Chem. Inf. Model 2003 43 429 434 10.1021/ci025590u 12653505

[92] Hou TJ Xia K Zhang W Xu XJ ADME evaluation in drug discovery. 4. Prediction of aqueous solubility based on atom contribution approach J. Chem. Inf. Model 2004 44 266 275 10.1021/ci034184n 14741036

[93] Huuskonen J Estimation of aqueous solubility for a diverse set of organic compounds based on molecular topology J. Chem. Inf. Model 2000 40 773 777 10.1021/ci9901338 10850781

